# Sexual Dimorphism in Doxorubicin-induced Systemic Inflammation: Implications for Hepatic Cytochrome P450 Regulation

**DOI:** 10.3390/ijms21041279

**Published:** 2020-02-14

**Authors:** Marianne K.O. Grant, Ibrahim Y. Abdelgawad, Christine A. Lewis, Beshay N. Zordoky

**Affiliations:** Department of Experimental and Clinical Pharmacology, University of Minnesota College of Pharmacy, Minneapolis, MN 55455, USAabdel217@umn.edu (I.Y.A.); lewi1050@umn.edu (C.A.L.)

**Keywords:** Doxorubicin, inflammation, sexual dimorphism, cytochrome P450, liver

## Abstract

Doxorubicin (DOX) is an effective chemotherapeutic agent used to treat a wide variety of malignancies. In addition to its multi-organ toxicity, DOX treatment has been shown to induce systemic inflammation in patients and experimental animals. Inflammation alters the expression of hepatic cytochrome P450 (CYP) enzymes, which play important roles in drug metabolism and DOX-induced toxicity. Significant sex differences have been reported in DOX-induced toxicity; however, sex differences in DOX-induced systemic inflammation and the potential effects on hepatic CYP expression have not been determined. In the current work, male and female C57Bl/6 mice were administered DOX (20 mg/kg by intraperitoneal injection), and groups of mice were sacrificed 24 and 72 h after DOX administration. DOX elicited a systemic inflammatory response in both male and female mice, but the inflammatory response was stronger in male mice. DOX altered the expression of hepatic CYP isoforms in a sex-dependent manner. Most notably, inhibition of Cyp2c29 and Cyp2e1 was stronger in male than in female mice, which paralleled the sex differences in systemic inflammation. Therefore, sex differences in DOX-induced systemic inflammation may lead to sexually dimorphic drug interactions, in addition to contributing to the previously reported sexual dimorphism in specific DOX-induced organ toxicity.

## 1. Introduction

Doxorubicin (DOX) is an effective chemotherapeutic agent used to treat a wide variety of solid and hematological malignancies. Nevertheless, the clinical utility of DOX is limited by its multi-organ toxicity. DOX is known for its cardiotoxic effects that may lead to cardiac dysfunction and heart failure [1]. In addition to cardiotoxicity, DOX has also been shown to cause hepatotoxicity [2,3,4,5,6], gastro-intestinal toxicity [7], and nephrotoxicity [8,9]. Although DOX-induced toxicity is likely to be multi-factorial, inflammation has been shown to be a central player in mediating DOX-induced toxicity [10,11,12,13,14]. In addition to organ-specific inflammatory changes, DOX treatment has been shown to cause systemic inflammation in cancer patients [10], piglets [7], and rodents [15].

We and others have shown that inflammation plays a major role in the regulation of hepatic cytochrome P450 (CYP) enzymes [16,17,18]. Since hepatic CYP enzymes are the main drug-metabolizing enzymes, DOX-induced inflammation may lead to significant changes in drug metabolism and drug interactions. Indeed, we and others have previously demonstrated that acute DOX toxicity alters the expression of several hepatic CYP isoforms in male rats [5,19], which resulted in altered metabolism of clinically relevant drugs [19,20]. Importantly, DOX/cyclophosphamide chemotherapy caused a differential effect on drug-metabolizing enzyme activities in breast cancer patients with a notable reduction of CYP2C9 activity by 315% [21].

In addition to their important role in drug metabolism, CYP enzymes play important toxicological roles. For instance, CYP enzymes metabolize arachidonic acid to different hydroxyeicosatetraenoic acid and epoxyeicosatrienoic acid metabolites [22]. These CYP-mediated endogenous metabolites, among others, play important roles in modulating DOX-induced toxicity [5,23]. Therefore, determining the effect of DOX administration on hepatic CYP enzymes will have a paramount impact on our understanding of potential DOX-induced drug interactions as well as DOX-induced toxicity.

We have recently shown that acute DOX administration caused a sex-dependent alteration in the gene expression of several *CYP* isoforms in the heart of C57Bl/6 mice [11]. Since all previous studies reporting the effect of DOX on hepatic CYP expression and activity were performed using male experimental animals [5,19], sex-related differences in the hepatic effects of DOX have not been identified. Therefore, in the current study, we determined the effect of acute DOX administration on the expression of hepatic CYP enzymes in male and female C57Bl/6 mice to reveal potential sex-related differences.

## 2. Results

### 2.1. Effect of DOX Treatment on Inflammatory Markers

**Figure 1:**
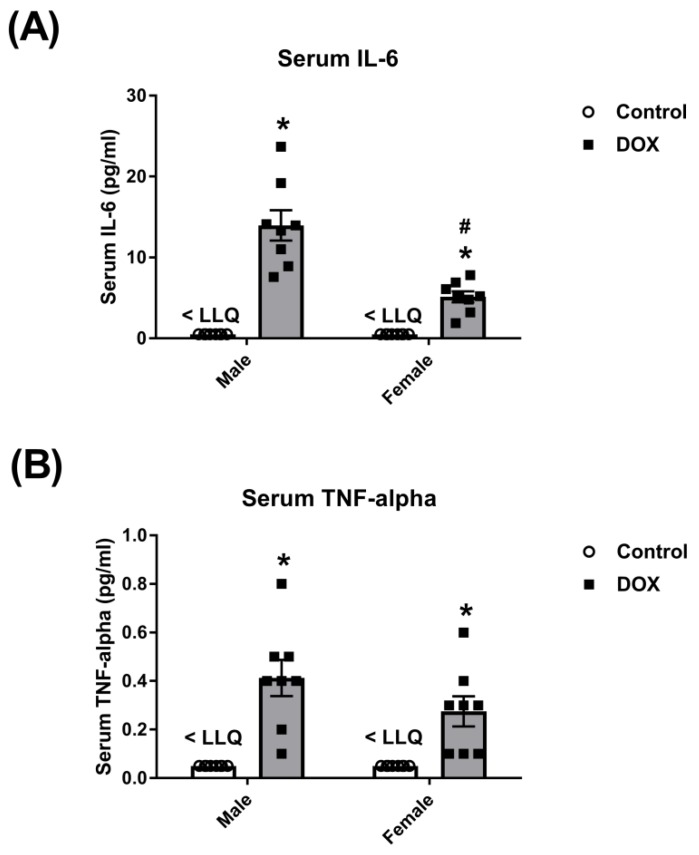
Effect of DOX treatment on the serum level of inflammatory markers. Serum was collected from male or female C57Bl/6 mice 24 h following the administration of a single intraperitoneal injection of 20 mg/kg DOX or saline (*n* = 8 per group). Inflammatory markers (**A**) IL-6 and (**B**) TNF-alpha were measured using the Luminex platform. Data are presented as the mean ± SEM. * *p* < 0.05, compared to saline-treated mice of the same sex; # *p* < 0.05, compared to male DOX-treated mice.

We and others have demonstrated significant sex-related differences in DOX-induced cardiotoxicity and nephrotoxicity [11,22,24,25]. Nevertheless, sex-related differences in DOX-induced systemic inflammation have not been previously reported. Therefore, we determined the effect of acute DOX administration on serum level of key inflammatory mediators, interleukin 6 (IL-6) and tumor necrosis factor-alpha (TNF-alpha), in adult male and female C57Bl/6 mice 24 h post-treatment. Acute DOX administration (20 mg/kg) elicited a systemic inflammatory response and caused a significantly higher increase in the serum level of IL-6 in male than in female mice (14 pg/mL in DOX-treated males vs. 5 pg/mL in DOX-treated females). Although the TNF-alpha serum level was higher in DOX-treated male than female mice (0.4 pg/mL in DOX-treated males vs. 0.3 pg/mL in DOX-treated females), this difference was not statistically significant. The serum levels of IL-6 and TNF-alpha were below the lower limit of quantification (LLQ) in control male and female mice (Figure 1).

**Figure 2:**
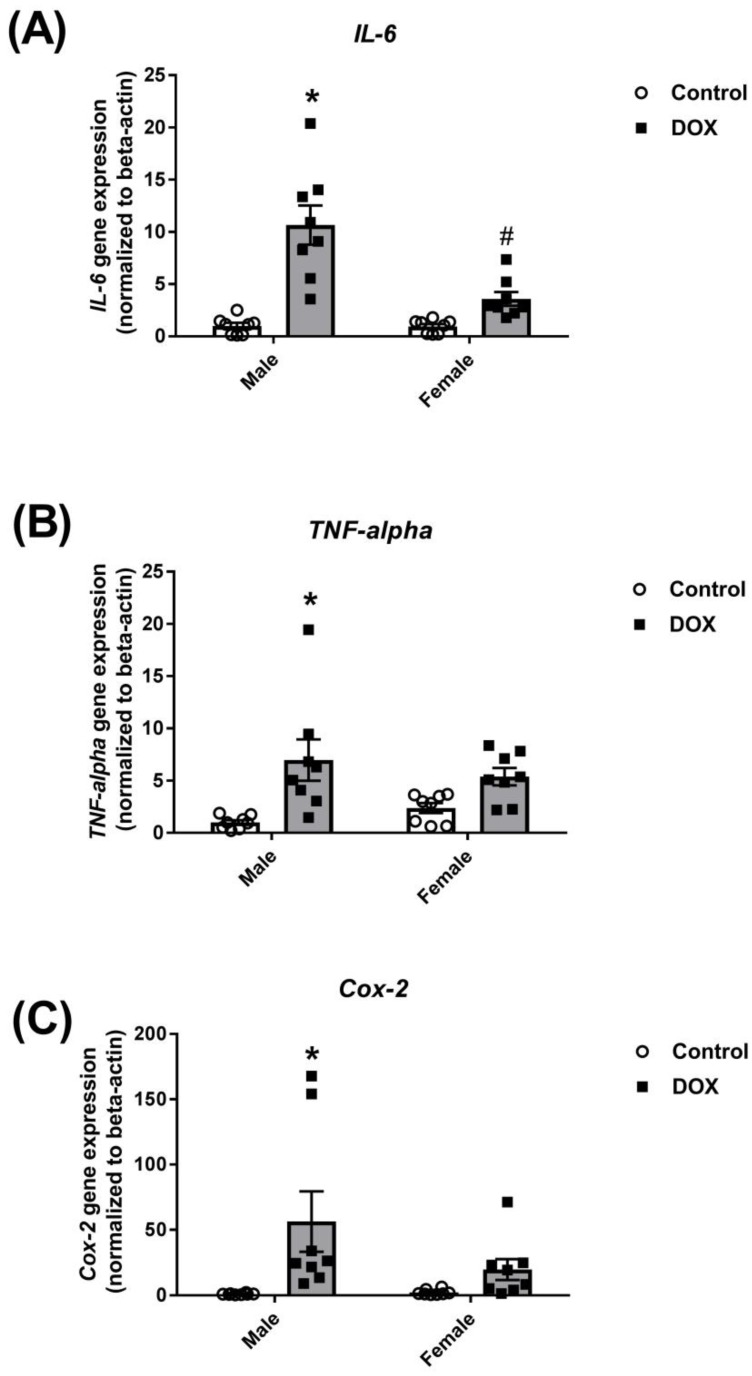
Effect of DOX treatment on the gene expression of inflammatory markers in the liver. Livers were collected from male or female C57Bl/6 mice 24 h following the administration of a single intraperitoneal injection of 20 mg/kg DOX or saline, and total RNA was isolated (*n* = 8 per group). Gene expression of (**A**) *IL-6*, (**B**) *TNF-alpha*, or (**C**) *Cox-2* was determined by real-time PCR. Results were normalized to *beta-actin* and are expressed relative to the male control. Data are presented as the mean ± SEM. * *p* < 0.05, compared to control mice of the same sex; # *p* < 0.05, compared to male DOX-treated mice.

Corroborating these findings, acute DOX administration caused a significant 10-, 7-, and 60-fold induction of the inflammatory markers *IL-6*, *TNF-alpha*, and *cyclooxygenase-2* (*Cox-2*) gene expression, respectively, in the liver of male mice (Figure 2). There was a modest induction of these markers in the liver of female mice, but the differences between the female-control and female-DOX groups did not reach statistical significance (Figure 2).

### 2.2. Effect of DOX Treatment on Cyp1 Family Expression

**Figure 3:**
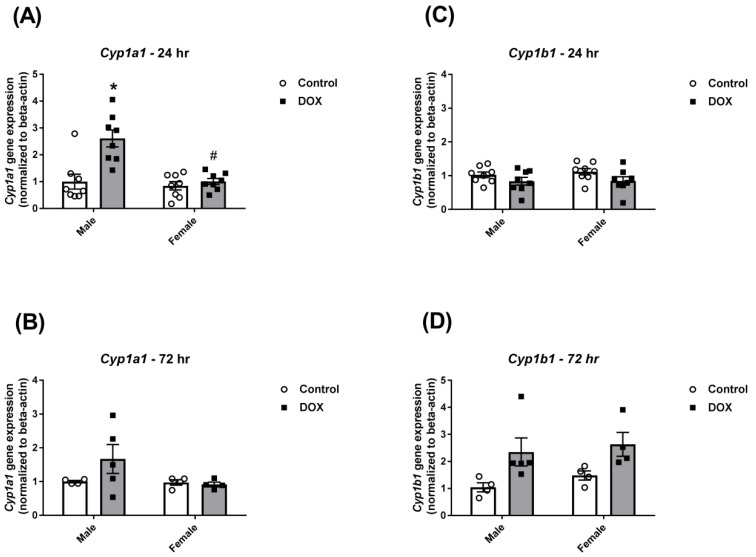
Effect of DOX treatment on the gene expression of the Cyp1 family in the liver. Livers were collected from male or female C57Bl/6 mice 24 h (*n* = 8 per group) or 72 h (*n* = 4–5 per group) following the administration of a single intraperitoneal injection of 20 mg/kg DOX or saline, and total RNA was isolated. Gene expression of *Cyp1a1* was determined by real-time PCR in samples collected (**A**) 24 or (**B**) 72 h following DOX treatment. Gene expression of *Cyp1b1* in samples collected (**C**) 24 or (**D**) 72 h following DOX treatment. Results were normalized to *beta-actin* and are expressed relative to the male control. Data are presented as the mean ± SEM. * *p* < 0.05, compared to control mice of the same sex; # *p* < 0.05, compared to male DOX-treated mice.

Twenty-four hours following DOX administration, DOX caused a significant 2.6-fold induction of hepatic *Cyp1a1* gene expression in male mice only (Figure 3A). Two-way ANOVA showed significant DOX and sex effects as well as a significant interaction between DOX exposure and sex (Table 1). Seventy-two hours following DOX administration, DOX-induced *Cyp1a1* upregulation in male mice was not statistically significant (Figure 3B). On the other hand, there was no significant change in *Cyp1b1* gene expression 24 h following DOX administration in male or female mice (Figure 3C). Nevertheless, there was a significant DOX effect on *Cyp1b1* induction after 72 h (Figure 3D and Table 1).

### 2.3. Effect of DOX Treatment on Cyp2 Family Expression

**Figure 4:**
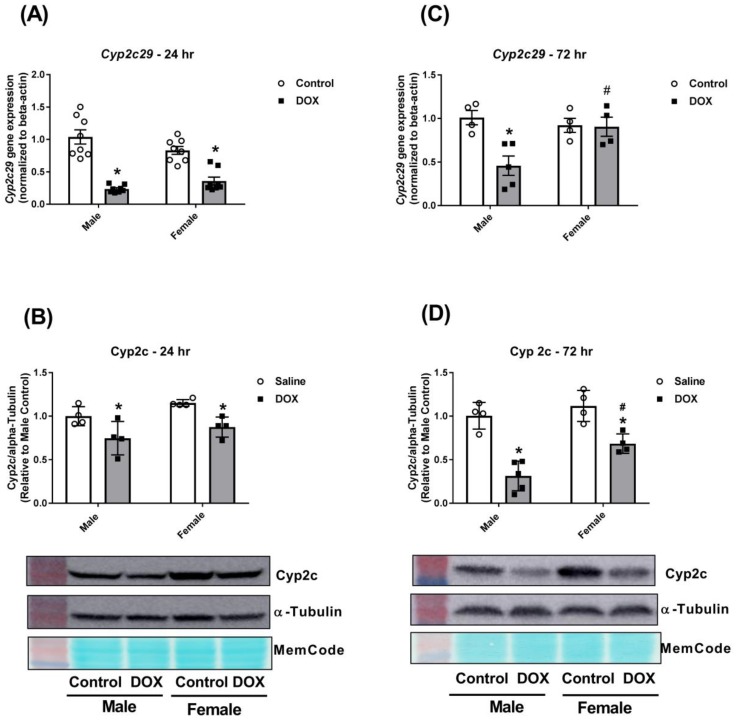
Effect of DOX treatment on the expression of the Cyp2c sub-family in the liver. Livers were collected from male or female C57Bl/6 mice 24 or 72 h following the administration of a single intraperitoneal injection of 20 mg/kg DOX or saline, and total proteins and RNA were isolated. (**A**) Gene expression of *Cyp2c29* was determined by real-time PCR (*n* = 8 per group) and (**B**) protein levels of total Cyp2c were determined by western blotting (*n* = 4 per group) in samples collected 24 h following DOX treatment. (**C**) Gene expression of *Cyp2c29* (*n* = 4–5 per group) and (**D**) protein levels of Cyp2c (*n* = 4–5 per group) were determined in samples collected 72 h following DOX treatment. PCR results were normalized to *beta-actin*, and protein expression was normalized to alpha-tubulin. Results of all groups are expressed relative to the male control. Data are presented as the mean ± SEM. * *p* < 0.05, compared to control mice of the same sex; # *p* < 0.05, compared to male DOX-treated mice.

DOX caused a significant 75% and 50% inhibition in *Cyp2c29* gene expression in male and female mice 24 h after administration, respectively (Figure 4A). Measurement of Cyp2c protein expression corroborated the gene expression results. There was a significant 25% and 20% inhibition of Cyp2c protein expression 24 h after DOX administration in male and female mice, respectively (Figure 4B). Interestingly, *Cyp2c29* gene expression returned to the control value in females, but not in male mice, 72 h after DOX administration (Figure 4C). Two-way ANOVA showed a significant DOX effect as well as a significant interaction between DOX exposure and sex (Table 1). At the protein expression level, DOX caused 70% inhibition of Cyp2c protein expression in male mice, but only 30% inhibition in female mice, 72 h after DOX administration (Figure 4D). There was a significant difference in Cyp2c protein expression between DOX-treated male and female mice (Figure 4D).

**Figure 5:**
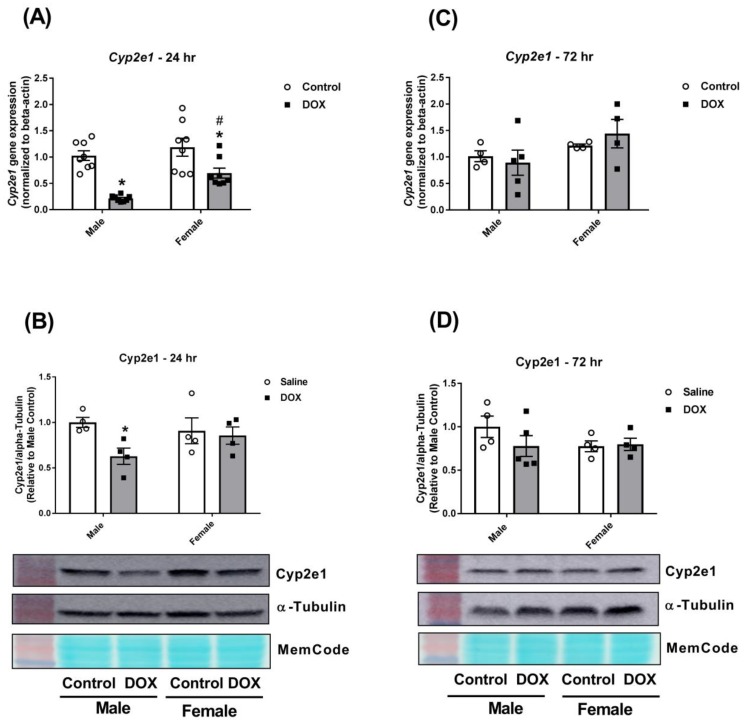
Effect of DOX treatment on the expression of Cyp2e1 in the liver. Livers were collected from male or female C57Bl/6 mice 24 or 72 h following the administration of a single intraperitoneal injection of 20 mg/kg DOX or saline, and total proteins and RNA were isolated. (**A**) Gene expression of *Cyp2e1* was determined by real-time PCR (*n* = 8 per group), and (**B**) protein levels of Cyp2e1 were determined by western blotting (*n* = 4 per group) in samples collected 24 h following DOX treatment. (**C**) Gene expression of *Cyp2e1* (*n* = 4–5 per group) and (**D**) protein levels of Cyp2e1 (*n* = 4–5 per group) were determined in samples collected 72 h following DOX treatment. PCR results were normalized to *beta-actin*, and protein expression was normalized to alpha-tubulin. Results of all groups are expressed relative to the male control. Data are presented as the mean ± SEM. * *p* < 0.05, compared to control mice of the same sex; # *p* < 0.05, compared to male DOX-treated mice.

DOX inhibited *Cyp2e1* gene expression in both male and female mice 24 h after administration. However, there was significantly more inhibition in male (80% inhibition) than in female mice (30% inhibition) (Figure 5A). The marked inhibition of *Cyp2e1* gene expression in male mice resulted in a significant 38% inhibition in Cyp2e1 protein expression in male mice only (Figure 5B). There were no significant changes in *Cyp2e1* gene or protein expressions 72 h after DOX administration (Figure 5C,D).

### 2.4. Effect of DOX Treatment on Cyp4a Sub-family Expression

**Figure 6:**
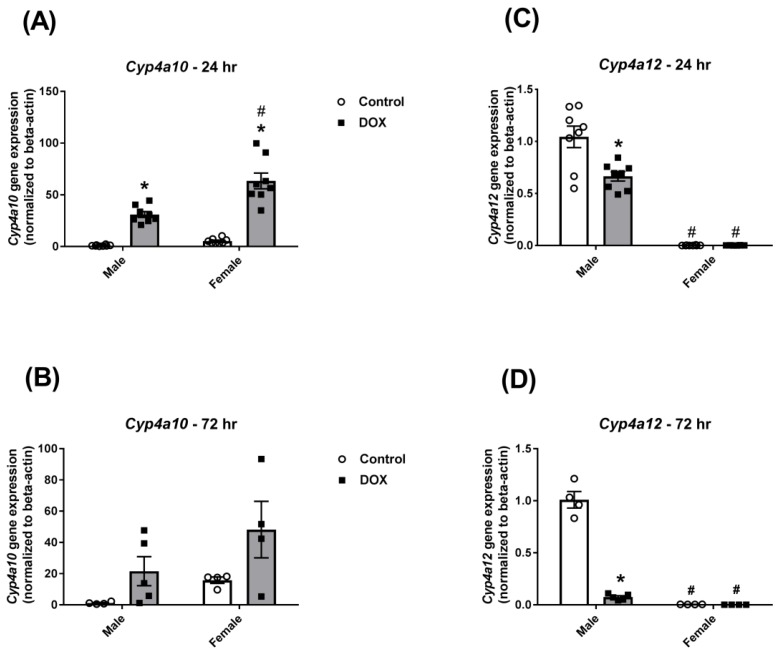
Effect of DOX treatment on the gene expression of the Cyp4a sub-family in the liver. Livers were collected from male or female C57Bl/6 mice 24 h (*n* = 8 per group) or 72 h (*n* = 4–5 per group) following the administration of a single intraperitoneal injection of 20 mg/kg DOX or saline, and total RNA was isolated. Gene expression of *Cyp4a10* was determined by real-time PCR in samples collected (**A**) 24 or (**B**) 72 h following DOX treatment. Gene expression of *Cyp4a12* in samples collected (**C**) 24 or (**D**) 72 h following DOX treatment. Results were normalized to *beta-actin* and are expressed relative to the male control. Data are presented as the mean ± SEM. * *p* < 0.05, compared to control mice of the same sex; # *p* < 0.05, compared to male mice of the same treatment.

DOX caused a significant 30-fold induction in *Cyp4a10* gene expression in male mice 24 h after administration (Figure 6A). Basal *Cyp4a10* gene expression was 5 times higher in females than males and was further induced 12-fold by DOX administration to be 60 times higher than its value in the control-male group (Figure 6A). Similar results were observed 72 h following acute DOX administration; nevertheless, the differences did not reach statistical significance (Figure 6B). *Cyp4a12* was expressed in male mice at much higher levels than in female mice (Figure 6C,D), confirming that *Cyp4a12* is a male-specific isoform as previously reported [24]. Interestingly, DOX caused a significant 35% and 90% inhibition of *Cyp4a12* gene expression after 24 h and 72 h of administration, respectively, in male mice (Figure 6C,D).

## 3. Discussion

The current study demonstrates, for the first time, sex-related differences in DOX-induced systemic inflammation and DOX-induced alteration of hepatic CYP expression in mice. DOX-induced systemic inflammation is a clinically relevant adverse effect of DOX treatment since it has been reported in patients receiving DOX-based chemotherapy [10]. Furthermore, systemic inflammatory reaction is associated with poor outcomes in cancer patients [25], which may be attributed to the role of interleukins in mediating progression, metastatic processes, and drug resistance of cancer cells [26]. Indeed, nutraceuticals with anti-inflammatory properties such as lycopene, curcumin, and resveratrol have been shown to reduce the toxicity and improve the efficacy of chemotherapeutic agents [14,27]. We and others have reported significant sex-related differences in acute and chronic DOX-induced cardiotoxicity and nephrotoxicity in experimental animals [11,28,29,30,31,32]. Sexual dimorphism of acute DOX-induced cardiotoxicity is associated with higher expression of inflammatory genes in the heart of male than female mice [11]. Similarly, sexual dimorphism of chronic DOX-induced cardiotoxicity is associated with higher inflammatory cell infiltration in male than in female hearts [30,32]. In the current study, we demonstrate significant sexual dimorphism in DOX-induced systemic inflammation. In agreement with previous studies [10], acute DOX administration elicited a systemic inflammatory response in both male and female mice; however, the inflammatory reaction was stronger in male mice. The observed sexual dimorphism in DOX-induced systemic inflammation may contribute to the previously reported sex differences in DOX-induced organ-specific toxicity.

In addition to a potential role in mediating sexual dimorphism of DOX-induced toxicity, sex differences in DOX-induced systemic inflammation may also lead to sex differences in altering the expression of hepatic CYP enzymes by DOX. We previously demonstrated significant sexual dimorphism in DOX-induced alteration of *CYP* gene expression in the heart of C57Bl/6 mice [11]. In the current work, we observed a male-specific induction of *Cyp1a1* gene expression 24 h after DOX administration. We previously reported that DOX upregulated *CYP1A1* gene expression in H9c2 cardiomyoblasts [33] and in hearts of male Sprague Dawley rats [23], an observation that was later confirmed by other investigators [34,35]. Since inflammation has been shown to downregulate *Cyp1a1* gene expression [16,36,37], the observed DOX-induced upregulation of the *Cyp1a1* gene in the current work is likely mediated by mechanisms other than inflammation. Indeed, DOX has been shown to activate the aryl hydrocarbon receptor (AhR), the main transcription factor regulating Cyp1 family gene expression [34]. We also observed a significant DOX effect to upregulate *Cyp1b1* gene expression 72 h following DOX administration in both male and female mice. In contrast to *Cyp1a1*, inflammation has been shown to upregulate *Cyp1b1* [16,38]. Therefore, the observed DOX-induced upregulation of the *Cyp1b1* gene can likely be attributed to both inflammation and AhR activation.

With regard to the Cyp2 family, acute DOX administration caused a marked inhibition of *Cyp2c29* isoform gene expression and total Cyp2c protein expression in the liver of male and female mice. Nevertheless, DOX caused a stronger inhibition in male than in female mice 72 h following DOX administration. Importantly, CYP2C9 enzyme activity was reduced by 319% in breast cancer patients who were treated by DOX/cyclophosphamide therapy. Cyp2c enzymes are known to be down-regulated by inflammation [16,17,18,37]. Therefore, the observed sexually dimorphic downregulation is likely to be attributed to the sex difference in the DOX-induced inflammatory reaction. Since Cyp2c enzymes are known to metabolize arachidonic acid to epoxyeicosatrienoic acids [39], inhibition of Cyp2c enzymes may lead to a reduction in the level of these anti-inflammatory molecules, thus worsening DOX-induced inflammation via a positive feedback loop. Interestingly, inhibition of Cyp2c by DOX was more marked 72 h after administration than at the 24-hour time-point, demonstrating an exaggeration of the DOX effect over time. In contrast, DOX caused male-specific inhibition of the Cyp2e1 enzyme that was completely recovered at the 72-hour time-point. Downregulation of Cyp2e1 by DOX can also be attributed to DOX-induced inflammation since inflammation has been shown to downregulate hepatic Cyp2e1 [16].

Cyp4a is a family of enzymes that play an important role in the metabolism of endogenous compounds, e.g., arachidonic acid [22]. We previously demonstrated that acute DOX toxicity induced CYP4A enzymes in the liver of male Sprague Dawley rats [5]. In agreement with this study, DOX caused a marked induction of *Cyp4a10* gene expression in both male and female mice in the current work. Lipopolysaccharide-induced inflammation has also been shown to upregulate hepatic Cyp4a enzymes in male rats [16]. In the current study, DOX caused a significant inhibition in *Cyp4a12* gene expression in male mice. Since *Cyp4a12* is a male-specific isoform [24], its inhibition may be attributed to DOX-induced perturbation in testosterone levels secondary to DOX-induced gonadotoxicity as we previously demonstrated [28]. In support of this argument, castration has been shown to inhibit Cyp4a protein expression and activity in male mice, an effect that was reversed by testosterone supplementation [40]. Further research is warranted to delineate the potential impact of DOX-induced gonadotoxicity on the regulation of sexually dimorphic CYP enzymes.

## 4. Materials and Methods

### 4.1. Animals

All experimental procedures involving animals were approved by the University of Minnesota Institutional Animal Care and Use Committee (IACUC number A3456-01; Protocol number 1508-32853A approved on 10/02/2015). Male (*n* = 26) and female (*n* = 24) C57Bl/6 mice were purchased from Charles River Laboratories (Raleigh, NC, USA) at twelve weeks of age. After an acclimation period of one week, mice were administered either 20 mg/kg DOX by intraperitoneal (IP) injection (DOX group) or an equivalent volume of sterile normal saline (control group) as we previously described [11,28]. Thereafter, mice were humanely euthanized at 24 h (8 male-control, 8 male-DOX, 8 female-control, and 8 female-DOX) or at 72 h (4 male-control, 5 male-DOX, 4 female-control, and 4 female-DOX) following DOX or saline administration. Mortality was observed in the male-DOX group followed for 72 h (1 out of 6 male-DOX mice) as previously reported in our earlier studies utilizing this model [11,28]. At the experimental end point, mice were euthanized by decapitation under isoflurane anesthesia. Terminal blood was collected, and livers were harvested, washed in ice-cold phosphate buffered saline solution, flash frozen in liquid nitrogen, and stored at −80 °C until further analysis.

### 4.2. Measurement of Inflammatory Markers

Terminal blood was collected from animals euthanized 24 h following DOX or saline administration and incubated at room temperature for 20 min to allow blood to clot. Samples were centrifuged at 4000 rpm for 30 min at 4 °C; serum was collected and stored at −80 °C until use. Serum samples were analyzed by the Cytokine Reference Laboratory (University of Minnesota) for mouse-specific IL-6 and TNF-alpha using the Luminex platform and done as a multiplex. The magnetic bead set (catalog LXSAMSM-03) was purchased from R&D Systems (Minneapolis, MN, USA). Samples were assayed according to manufacturer’s instructions. Briefly, fluorescent color-coded beads coated with a specific capture antibody were added to each sample. After incubation and washing, biotinylated detection antibody was added followed by phycoerythrin-conjugated streptavidin. The beads were read on a Luminex instrument (Bioplex 200, Bio-Rad Laboratories, Inc., Hercules, CA, USA), which is a dual-laser fluidics-based instrument. Samples were run in duplicate, and values were interpolated from 5-parameter fitted standard curves.

### 4.3. RNA Extraction and Real-time PCR

Total RNA from the frozen liver tissues was isolated using TRIzol^®^ reagent (Life Technologies, Carlsbad, CA, USA), according to the manufacturer’s instructions, and quantified by using a Nanodrop 8000 spectrophotometer (Thermo Fisher Scientific, Wilmington, DE, USA). Thereafter, 1.5 μg of total RNA from each sample was used to synthesize first-strand cDNA by the high-capacity cDNA reverse transcription kit (Life Technologies, Carlsbad, CA, USA) according to the manufacturer’s instructions. Real time-polymerase chain reaction (PCR) was used to measure specific mRNA expression by subjecting the resulting cDNA to PCR amplification using 384-well optical reaction plates in an ABI 7900HT instrument (Applied Biosystems, Foster City, CA, USA). The 20 μL reaction mix contained 1 μL of cDNA sample, 0.025 μL of 30 μM forward primer and 0.025 μL of 30 μM reverse primer (40 nM final concentration of each primer), 10 μL of SYBR Green Universal Mastermix (Life Technologies, Carlsbad, CA, USA), and 8.95 μL of nuclease-free water as we described previously. Since we previously reported sex-dependent changes of certain *CYP* genes by acute DOX exposure in hearts of C57Bl/6 mice [11], these *CYP* genes were selected for the current study. The primers used in the current study were chosen from previously published studies, checked with the Primer-BLAST on-line tool, and are listed in Table 2. Thermocycling conditions were initiated at 95 °C for 10 min, followed by 40 PCR cycles of denaturation at 95 °C for 15 sec, and annealing/extension at 60 °C for 1 min. To ensure the specificity of the primers and the purity of the final PCR product, melting curve analysis was performed at the end of each cycle.

### 4.4. Protein Extraction and Western Blotting

Frozen liver tissues were homogenized as described previously, and protein concentration was determined using the Pierce bicinchoninic acid (BCA) protein assay kit according to manufacturer’s instructions (Pierce, Thermo Fisher Scientific, Rockford, IL, USA). Thereafter, 50 μg of protein per sample was separated by sodium dodecyl sulfate-polyacrylamide gel electrophoresis (SDS-PAGE) and electro-transferred onto nitrocellulose membranes as we previously described [41]. Primary rabbit antibodies against CYP2C and CYP2E1 (catalog ab137015 and ab151544; 1:1000 dilution) were purchased from Abcam (Cambridge, MA, USA). Primary rabbit antibody against alpha-tubulin (catalog 2144; 1:1000 dilution) was purchased from Cell Signaling Technology (Danvers, MA, USA). Secondary anti-rabbit conjugated to HRP was purchased from Jackson ImmunoResearch (catalog 111-035-144; 1:10,000 dilution; West Grove, PA, USA). Band intensities were quantified using ImageJ software (National Institutes of Health, Bethesda, MD, USA). Alpha-tubulin protein levels were used as loading controls to normalize the band intensities.

### 4.5. Statistical Analysis

Statistical analysis and data presentation were performed using GraphPad Prism software (version 7.04) for Windows, La Jolla California USA, www.graphpad.com. Data are presented as the mean ± SEM. Comparisons among different sex and treatment groups were performed by 2-way analysis of variance (ANOVA), followed by Tukey’s multiple comparison tests. A *p* value of < 0.05 was taken to indicate statistical significance.

## 5. Conclusions

Acute DOX administration elicits a systemic inflammatory response in a sex-dependent manner. Male mice are more susceptible to DOX-induced inflammation, which may contribute to the previously reported sexual dimorphism of DOX-induced cardiotoxicity and nephrotoxicity. Importantly, sex differences in DOX-induced inflammation may also cause sex-dependent alteration of hepatic CYP enzymes. Nevertheless, other mechanisms may also contribute to the alteration of hepatic CYP by acute DOX administration, including AhR activation and perturbation in sex hormones. Further research is needed to delineate the possible contribution of these factors to the sexual dimorphism of DOX-induced alteration of hepatic CYP expression.

## Figures and Tables

**Table 1:** Two-Way ANOVA for *CYP* Gene Expression Data.

	**24 h Post-DOX**					
	**DOX Effect**		**Sex Effect**		**Interaction**	
	**F (1, 28)**	***p* Value**	**F (1, 28)**	***p* Value**	**F (1, 28)**	***p* Value**
***Cyp1a1***	14.75	0.0006	14.61	0.0007	9.855	0.004
***Cyp1b1***	ns		ns		ns	
***Cyp2c29***	82.54	<0.0001	ns		5.58	0.0254
***Cyp2e1***	36.63	<0.0001	8.817	0.0061	ns	
***Cyp4a10***	114.9	<0.0001	20.01	0.0001	11.67	0.002
***Cyp4a12***	11.42	0.0022	229.9	<0.0001	11.45	0.0021
	**72 h Post-DOX**					
	**DOX Effect**		**Sex Effect**		**Interaction**	
	**F (1, 13)**	***p* Value**	**F (1, 13)**	***p* Value**	**F (1, 13)**	***p* Value**
***Cyp1a1***	ns		ns		ns	
***Cyp1b1***	9.828	0.0079	ns		ns	
***Cyp2c29***	8.005	0.0142	ns		7.136	0.0192
***Cyp2e1***	ns		ns		ns	
***Cyp4a10***	6.711	0.0224	ns		ns	
***Cyp4a12***	153.1	<0.0001	202.6	<0.0001	151.4	<0.0001

**Table 2:** Primers used in this study.

Gene	Forward Primer (5′–3′)	Reverse Primer (5′–3’)
*IL-6*	CCA GAG ATA CAA AGA AAT GAT GG	ACT CCA GAA GAC CAG AGG AAA T
*TNF-alpha*	CCA GAC CCT CAC ACT CAG ATC A	CAC TTG GTG GTT TGC TAC GAC
*Cox-2*	CTG GTG CCT GGT CTG ATG ATG	GGC AAT GCG GTT CTG ATA CTG
*Cyp1a1*	GGT TAA CCA TGA CCG GGA ACT	TGC CCA AAC CAA AGA GAG TGA
*Cyp1b1*	AAT GAG GAG TTC GGG CGC ACA	GGC GTG TGG AAT GGT GAC AGG
*Cyp2c29*	TGG TCC ACC CAA AAG AAA TTG A	GCA GAG AGG CAA ATC CAT TCA
*Cyp2e1*	CCC AAG TCT TTA ACC AAG TTG GC	CTT CCA TGT GGG TCC ATT ATT GA
*Cyp4a10*	GTG CTG AGG TGG ACA CAT TCA T	TGT GGC CAG AGC ATA GAA GAT C
*Cyp4a12*	TGA CCC CAG CTT TCC ACT ATG	TTG TTC AGG TCC TCA ACT GCC
*Beta-actin*	TAT TGG CAA CGA GCG GTT CC	GGC ATA GAG GTC TTT ACG GAT GTC

## Abbreviations

AhR	Aryl hydrocarbon Receptor
Cox-2	Cyclooxygenase-2
CYP	Cytochrome P450
DOX	Doxorubicin
IL-6	Interleukin-6
TNF-alpha	Tumor Necrosis Factor-alpha
